# Genomic Characterization of *Salmonella* Typhimurium Isolated from Guinea Pigs with Salmonellosis in Lima, Peru

**DOI:** 10.3390/microorganisms10091726

**Published:** 2022-08-27

**Authors:** Dennis E. Carhuaricra Huaman, Luis R. Luna Espinoza, Carmen L. Rodríguez Cueva, Carla G. Duran Gonzales, Raúl H. Rosadio Alcántara, João C. Setubal, Lenin Maturrano Hernández

**Affiliations:** 1 Research Group in Biotechnology Applied to Animal Health, Production and Conservation (SANIGEN), Laboratory of Biology and Molecular Genetics, Faculty of Veterinary Medicine, Universidad Nacional Mayor de San Marcos, San Borja, Lima 15021, Peru; 2 Programa de Pós-Graduação Interunidades em Bioinformática, Instituto de Matemática e Estatística, Universidade de São Paulo, Rua do Matão 1010, São Paulo CEP 05508-090, SP, Brazil; 3 Departamento de Bioquímica, Instituto de Química, Universidade de São Paulo, Av. Prof. Lineu Prestes, 748-Cidade Universitária, São Paulo CEP 05508-000, SP, Brazil

**Keywords:** *Salmonella* Typhimurium, guinea pig, comparative genomics, antimicrobial resistance genes

## Abstract

*Salmonella enterica* subsp. *enterica* serovar Typhimurium (*S.* Typhimurium) is one of the most important foodborne pathogens that infect humans globally. The gastrointestinal tracts of animals like pigs, poultry or cattle are the main reservoirs of *Salmonella* serotypes. Guinea pig meat is an important protein source for Andean countries, but this animal is commonly infected by *S*. Typhimurium, producing high mortality rates and generating economic losses. Despite its impact on human health, food security, and economy, there is no genomic information about the *S*. Typhimurium responsible for the guinea pig infections in Peru. Here, we sequence and characterize 11 *S*. Typhimurium genomes isolated from guinea pigs from four farms in Lima-Peru. We were able to identify two genetic clusters (HC100_9460 and HC100_9757) distinguishable at the H100 level of the Hierarchical Clustering of Core Genome Multi-Locus Sequence Typing (HierCC-cgMLST) scheme with an average of 608 SNPs of distance. All sequences belonged to sequence type 19 (ST19) and HC100_9460 isolates were typed in silico as monophasic variants (1,4,[5],12:i:-) lacking the *fljA* and *fljB* genes. Phylogenomic analysis showed that human isolates from Peru were located within the same genetic clusters as guinea pig isolates, suggesting that these lineages can infect both hosts. We identified a genetic antimicrobial resistance cassette carrying the *ant(3)-Ia*, *dfrA15*, *qacE,* and *sul1* genes associated with transposons TnAs3 and IS21 within an IncI1 plasmid in one guinea pig isolate, while antimicrobial resistance genes (ARGs) for β-lactam (*bla*_CTX-M-65_) and colistin (*mcr-1*) resistance were detected in Peruvian human-derived isolates. The presence of a virulence plasmid highly similar to the pSLT plasmid (LT2 reference strain) containing the *spvRABCD* operon was found in all guinea pig isolates. Finally, seven phage sequences (STGP_Φ1 to STGP_Φ7) were identified in guinea pig isolates, distributed according to the genetic lineage (H50 clusters level) and forming part of the specific gene content of each cluster. This study presents, for the first time, the genomic characteristics of *S*. Typhimurium isolated from guinea pigs in South America, showing particular diversity and genetic elements (plasmids and prophages) that require special attention and also broader studies in different periods of time and locations to determine their impact on human health.

## 1. Introduction

*Salmonella* is one of the most important foodborne pathogens in humans, responsible for 78 million illnesses with a balance of 59,000 deaths each year [1]. *Salmonella enterica* consists of more than 2600 serovars and *S. enterica* serovar Typhimurium is one of the most frequently reported in human infections [2,3]. Poultry, pigs, and cattle are the main reservoirs of *Salmonella* and constitute vehicles for human infections when there is the consumption of contaminated food [4]. In recent decades, the intensification of farm practices has led to the emergence of zoonotic pathogens that threaten animal and human health. The emergence of new variants of *S*. Typhimurium has been reported recently in pigs (U288, ST34) and humans (ST313) and poses a serious threat to health and food safety [5,6,7]. Furthermore, variants lacking one or both flagellar phases have been widely reported. The monophasic variants of *S.* Typhimurium (4,[5],12:i:-) emerged last century and multiple clones have been reported worldwide and are characterized by not expressing the second-phase flagellar antigen (defined as 1,2 in the antigenic formula) of *S.* Typhimurium (4,[5],12:i:1,2) [8,9]. Genomic surveillance provides an opportunity to identify variants, genetic signals of resistance and virulence, or host adaptation of pathogens such as Salmonella, facilitating the management of emerging variants that pose a health threat [10,11].

The guinea pig (*Cavia porcellus*) is a rodent domesticated in the Central Andes of South America 3000–6000 years ago and used by Andean populations as food source and for ritual and medicinal purposes [12]. From that period until today, guinea pig farming has been one of the most important economic and cultural practices for Andean populations [13]. In recent decades, guinea pig production has undergone an enormous transformation from small-scale family farming with a few dozen animals to large farms with tens of thousands of animals. As was observed in other pathogens, the intensification of farming practices has led to the emergence of host-adapted lineages [14,15]. In guinea pig farms, *Salmonella* is responsible for high morbidity and mortality affecting the economy and food security in Andean countries such as Peru; although there is no representative information about the regional prevalence of salmonellosis in its commercial production, a recent study performed in 40 animals from 3 farms in La Libertad-Peru found a prevalence of 27.5%, with a higher percentage of positive samples from commercial breeding [16,17]. Salmonellosis mainly affects the gastrointestinal tract of guinea pigs, with diarrhea as the most evident clinical manifestation; however, subclinical infection can occur when the guinea pig acts as a latent carrier capable of transmitting *Salmonella* to other animals and humans [18]. Recent studies have reported that *S.* Typhimurium is the main etiological agent isolated from cases of salmonellosis in guinea pigs [19]. Despite its impact on animal health and potential zoonotic source, there is no information on the lineage of *S.* Typhimurium that infects guinea pigs. The specific features of the livestock production system in low and middle income countries (LMIC), such as animal overcrowding, informal trade, and deficiencies in biosecurity and veterinary service access, promote the emergence of recurrent outbreaks, raising the risk of zoonotic infections and resistance pathogen emergence [20]. A one health approach is necessary to study the impact of guinea pig salmonellosis on the health of the Peruvian population using modern genomics tools. The objective of this work was to perform a genomic analysis of *S.* Typhimurium isolates obtained from guinea pigs in four farms of Lima-Peru and compare them with available genomic sequences of *S.* Typhimurium from human origin from Peru.

## 2. Methods

### 2.1. Bacterial Isolates

In this study we analyzed 11 *S.* Typhimurium isolates collected from guinea pigs reared in four different farms located in Lima, Peru, between 2015–2016 (Table S1). The isolates were recovered from liver samples obtained from salmonellosis clinical cases. *Salmonella* isolation was performed according to the International Organization for Standardization (ISO) 6579:2002. Liver tissue fragments were aseptically transferred to buffered peptone water and incubated at 37 °C for 18 h. Enriched cultures were transferred to Rappaport Vassiliadis Soya (RSV) broth and then incubated at 42 °C for 24 h. An aliquot was streaked onto XLD (Xylose Lysine Deoxycholate) agar and incubated at 37 °C for 24 h. Presumptive *Salmonella* colonies were confirmed as *S.* Typhimurium by PCR reaction using primers according to [21]. All isolates were stored at −80 °C in 25% glycerol diluted in BHI broth.

#### Whole Genome Sequencing and Assembly

Confirmed *S.* Typhimurium isolates were selected for sequencing. Genomic DNA was extracted and purified using the PureLink™ Genomic DNA Mini Kit (Invitrogen, Waltham, MA, USA) and concentration was measured using Qubit dsDNA HS assay (Invitrogen). For library preparation, 1 ng of DNA was required for the Nextera XT protocol, and subsequent sequencing was carried out using 2 × 250 bp reads on the Illumina Miseq platform (Illumina, San Diego, CA, USA) at the Laboratory of Molecular Genetics and Biology, Faculty of Veterinary Medicine, UNMSM. The *fastq* files were retrieved and evaluated using FastQC v0.11.9 [22], the trimming of low-quality reads was performed with Trimmomatic v0.39 [23] using the options LEADING:3 TRAILING:3 SLIDINGWINDOW:4:20 MINLEN:50, and de novo assembly was performed with SPAdes v3.14.1 [24]. The genome completeness and contamination parameters to evaluate the assembly quality were calculated with the checkM software [25]. Finally, the Prokaryotic Genome Annotation Pipeline (PGAP) version 14 April 2022.build6021 was used for annotation, using default parameters.

### 2.2. Phylogenetic and Genetic Diversity Analysis of S. Typhimurium from Guinea Pigs

In order to phylogenetically characterize *S.* Typhimurium genomes isolated from guinea pigs, we used 21 reference genomes representing different lineages of *S.* Typhimurium isolated from different hosts and countries and 5 genomes from Peruvian human isolates. These genomes were downloaded from the GenBank, and their information is detailed in Supplementary Table S2. We used Snippy v 4.6.0 (https://github.com/tseemann/snippy) (accessed on 1 June 2022). to generate a core genome alignment. The complete genome sequence of *S.* Typhimurium LT2 was used as a reference strain (Accession number: NC_003197.1). Gubbins v2.4.1 software [26] was run for five iterations to remove recombinant regions before the phylogenetic reconstruction. From the core-genome alignment, we reconstruct a maximum-likelihood tree using IQ-TREE v1.6.12 [27] based on a GTR nucleotide substitution model with 1000 bootstrap replicates. Tree visualization and annotation was created using ggtree v3.0.4 package [28]. We computed pairwise SNP distances between genomes from the core-genome alignment using SNP-dists v0.6 (https://github.com/tseemann/snp-dists) (accessed on 3 June 2022).

### 2.3. MLST, Serotype, Prophage, Plasmid, and Antibiotic Resistance Genes [ARGs] Profiles Prediction and Comparative Genomics

We used mlst v2.19.0 tool (https://github.com/tseemann/mlst) (accessed on 16 May 2022) to determine multilocus sequence type [MLST] using the *Salmonella enterica* scheme from PubMLST. Serovar prediction was performed using SeqSero2 in the default k-mer-based mode [29,30]. ARGs, virulence genes, and plasmid replicon types were annotated using the Resfinder, Virulence factor database [VFDB], and PlasmidFinder databases with the ABRICATE v. 1.0.1 tool (https://github.com/tseemann/abricate) (accessed on 10 June 2022). ABRICATE was run using a minimum DNA identity of 80% and a minimum coverage of 80%. We searched all genomes for phage elements using the PHASTER database [31]. We took the “intact” phage elements as defined by a phage score of >90 and “questionable” phage with score of >80. These sequences were checked manually and compared between genomes using BLASTN to evaluate sequence conservation. The genomic comparison was performed using BLAST Ring Image Generator (BRIG) [32]. The genetic contexts of ARGs detected in genomes were performed in R using the ggplot2 and gggenes R packages [33].

## 3. Results

### 3.1. Genomic Characteristics and Diversity of S. Typhimurium Isolates Obtained from Guinea Pigs

We sequenced the whole genome of 11 *S.* Typhimurium isolates from guinea pigs that died of salmonellosis in four farms in Lima-Peru, sampled between 2015 and 2016 (Table S1). The genome size of these eleven sequences ranged between 4.85–5.10 Mb and %GC content was between 52.08–52.22%. The assembly quality assessed with the completeness and contamination parameters from checkM software was >99% and <1% for all genomes, respectively (Table 1). *Insilico* MLST typing using *S. enterica* schema from pubMLST showed that all genomes from guinea pigs belong to the ST19 type, the most prevalent genotype in serovar Typhimurium. To obtain a good resolution on *S.* Typhimurium diversity on guinea pigs farms, the H100 level of the Hierarchical Clustering of Core Genome Multi-Locus Sequence Typing (HierCC-cgMLST) scheme from Enterobase was used to categorize our isolates. The 11 isolates were classified into two distinct HierCC-HC100 clusters: 9 isolates belonging to the HC100_9757 cluster and 2 to the HC100_9460 cluster.

A core-genome alignment of the 11 isolates from guinea pigs and the *S.* Typhimurium LT2 reference strain was used to construct a matrix of pairwise SNP distances to explore genetic diversity between the *S.* Typhimurium isolates (Figure S1). The SNP distance between the two clusters (HC100_9757 and HC100_9460) ranged from 590 to 623 SNPs with an average of 608 SNPs, compared to the distance between HC100_9757 and the LT2 reference strain (576 to 606 SNPs) and between H100_9460 and LT2 (478 and 471 SNPs), suggesting a large divergence between these two clusters of guinea pig isolates (Figure S1). We also evaluated the intra-H100-cluster diversity; whereas two HC100_9460 isolates were closely related (3 SNPs difference), the HC100_9757 cluster contains important diversity between isolates (2-200SNP). We observed two sub-clusters in HC100_9757 that corresponded to the HC50-67422 and HC50-9757 classification; the distance between these sub-clusters was 188-225 SNPs whereas intra-cluster distance was less than 100 SNPs (Figure S1).

Molecular serotyping using SeqSero2 software revealed that two HC100_9460 (SMVET11 and SMVET20) isolates lacked part of the *fljAB* operon sequence and were typed as monophasic variant (1,4,[5],12:i:-). Local inspection of the *fljAB* operon in HC100_9460 isolates shows that the *fljA* and *fljB* genes were replaced by a phage sequence that we named STGP_Φ2 (~90 kb). Instead, the other lineage identified in guinea pigs, HC100_9757, contained both intact genes (*fljA* and *fljB*) but an inversion in the *hin* gene was observed (See Figure S2).

### 3.2. Phylogenomic Analysis of S. Typhimurium Isolated in Peru

**Figure 1:**
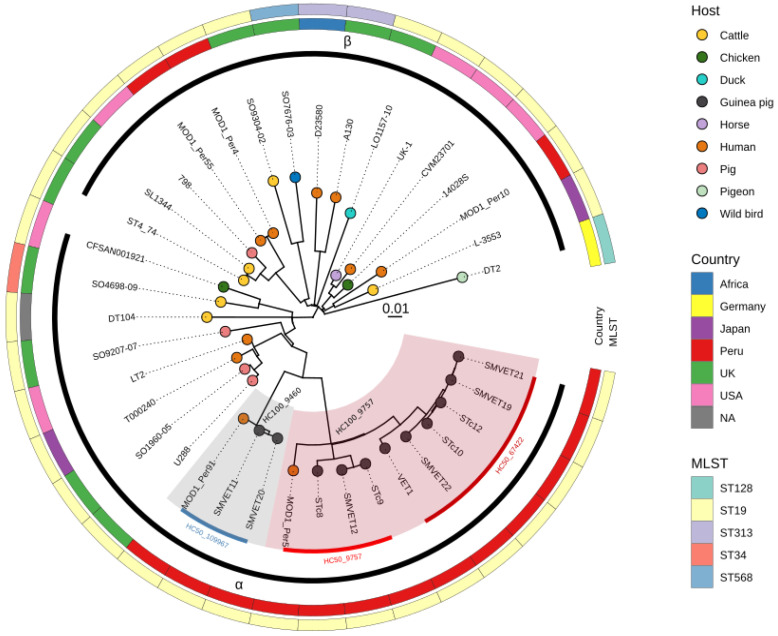
Phylogenetic tree of *S.* Typhimurium genome sequences isolated from guinea pigs in a context of 26 *S.* Typhimurium isolates from different hosts and locations including five sequences from Peruvian human sources. Two genetic clusters containing guinea pig isolates are highlighted in gray [HC100_9460] and red [HC100_9757]. The latter contains, in turn, two subclusters of H50: HC50-67422 and HC50-9757, that are represented by dark red and red bars, respectively.

We performed a phylogenomic analysis of 11 *S.* Typhimurium isolated from guinea pigs within a context of 22 additional *S.* Typhimurium complete genomes from diverse hosts and locations. We also included 5 genomes from human origins isolated in Peru that were downloaded from the GenBank database. The core-genome alignment length of the 37 genomes was 4,448,045 bp (90% of 4,951,383 bp LT2 reference strain). The phylogenetic tree constructed with 6015 SNPs in the core genome revealed two phylogroups [α and β] as described previously by [6]. All genomes from guinea pig origin were clustered into the α phylogroup, which includes isolates from humans and domestic animals (Figure 1). The two H100 clusters identified for guinea pig isolates (HC100_9757 and HC100_9460) are highlighted in the phylogenetic tree. These two lineages associated to guinea pigs were more distant to β phylogroups strains (>850 SNPs) and less distant from the LT2 reference strain (<600 SNPs) and phage type U288 (<670 SNPs); the SNP distance matrix of all genomes is depicted in Figure S3. Interestingly, two isolates from humans in Peru (sampled in 2012) also grouped within guinea pig lineages, suggesting that these lineages can infect both hosts (Figure 1). We were unable to obtain clinical information from Peruvian human isolates to confirm the source of contamination.

### 3.3. ARGs, Plasmid, and Virulence Factor Repertoire of S. Typhimurium from Guinea Pigs and Humans in Peru

**Figure 2:**
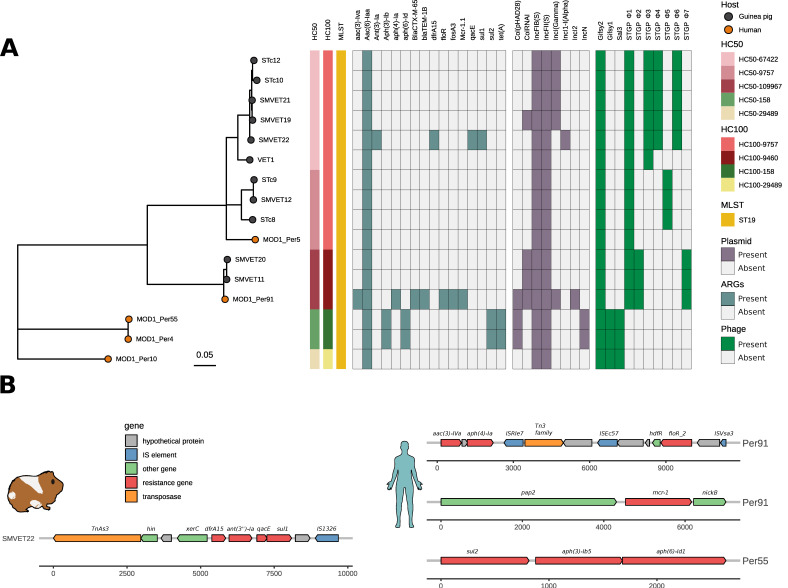
ARGs, plasmid, and phage profile in Peruvian *S.* Typhimurium isolates. (**A**) Phylogenomic tree based on SNP alignment of *S.* Typhimurium isolates including 11 from guinea pigs and 5 from human origin. Heatmap showing presence/absence of ARGs (blue), plasmid replicon (purple), and prophages (green). (**B**) Genetic context of ARGs detected in guinea pig and human isolates; the annotation of each gene in different colors is shown as per the key.

Since *S.* Typhimurium is an important human pathogen, we investigated the presence of ARGs and virulence genes in Peruvian isolates. We detected the chromosomal *aac(6′)-Iaa* gene for aminoglycoside resistance in all isolates from guinea pigs and humans. Surprisingly, the rest of the *S.* Typhimurium genomes from guinea pigs do not present other ARGs except the SMVET22 isolate that contains a resistance gene cassette with four ARGs: *ant(3′)-Ia*, *dfrA15*, *qacE,* and *sul1*, that confer resistance to aminoglycoside, trimetoprim, quaternary ammonium, and sulfamide, respectively (Figure 2A). This antimicrobial resistance cassette was 9678 nucleotides in length and inserted into an IncI1 plasmid of ~90 kb. We detected transposases flanking these resistance genes: IS1326 and TnAs3, belonging to the IS21 and Tn3 families, respectively (Figure 2B). 

Three Peruvian human isolates contained at least four ARGs, including the β-lactam resistant genes *bla*_CTX-M-65_ and *bla*_TEM-1B_ and the *mcr-1* colistin resistant gene in the MOD1_Per91 isolate. We detected four mobile genetic elements (MGEs) in the context of the *aac(3)-IVa*, *aph(4)-Ia,* and *floR* resistance genes; ISVsa3 and ISEc57 were flanking the *floR* gene (IS91 and IS21 family elements, respectively), while the ISRle7 (IS6 family element) and Tn3 family transposases were in the context of *aac(3)-IVa* and *aph(4)-Ia*. On the other hand, the *mcr-1* gene mobilized by an IncI2 plasmid was flanked by the *pap2-nikB* genes; we did not identify copies of IS*Apl1* in the neighborhood of the *mcr-1* gene (Figure 2B).

Eight types of plasmid replicons detected in Peruvian isolates belonged to the F type. All the analyzed genomes shared IncFII and IncFIB replicons; these correspond to a plasmid highly similar to the virulent plasmid pSLT from the LT2 reference strain, carrying the virulence operon (*spvRABCD*), but no ARGs. An Inclγ (Gamma) plasmid of ~93 Kb of size was detected in four guinea pig isolates of the cg-ST272 type; the annotations of this plasmid do not reveal the presence of any virulence or AR genes. On the other hand, Col (pHAD28), Incl2, and IncN plasmids were found in the human origin isolates, of which IncI2 detected in the MOD1_Per91 human isolate harbored the *mcr. 1.1* resistant gene (Table S3).

The *S.* Typhimurium genomes studied presented 144 different virulence genes associated with flagella, capsules, plasmids, adhesion systems, and type 3 secretion systems (T3SS) encoded in the pathogenicity islands SPI-1 and SP-2. All the genomes studied presented the main fimbrial operons necessary during tissue colonization: *fim* (encodes type I fimbriae), *lpf* (Long polar fimbriae), *bcf*, and the *csg* operon (Thin aggregative fimbriae—Tafi), except for the *pef* operon (Plasmid-encoded fimbriae), which was present in 81.1% of them. Additionally, the virulence genes located in the pSLT plasmid, involved in phenotypic virulence in rodents, were present in at least 29 of the genomes studied: *mig-14* (100%), *rck* (83.8%), and *spvBC* (78.4%). T3SS-related genes and pathogenicity islands (SPI-1 to SPI-5) were present in all studied genomes; these include: the *misL*, *sptP*, *avrA*, *pipB* genes; and the operons *inv*, *mgt*, *sic*, *sip*, *spa*, *ssa*, *ssc*, *sse*, *prg*, and *sic* (Figure S4).

### 3.4. Differential Gene Content in S. Typhimurium Isolates from Guinea Pigs Is Driven by Phages

**Figure 3:**
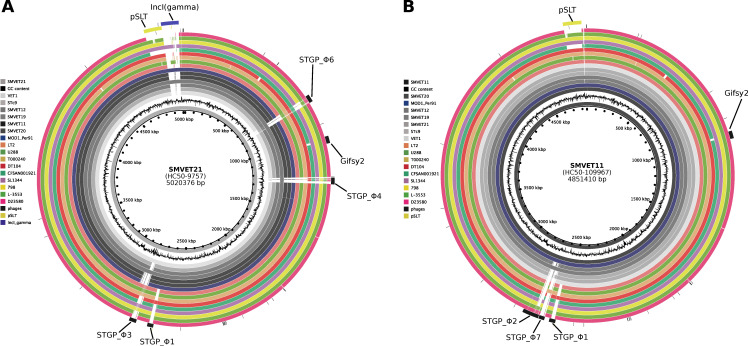
Comparative genomic showing differential prophage profiles in guinea pig isolates. Each ring represents the genome of a single *S.* Typhimurium isolated mapped to the (**A**) SMVET11 strain (H50-9757) or (**B**) SMVET11 strain (H50-109967); from the inner to the outermost ring, the first (innermost) ring shows the genome size in kbp, followed by SMVET21 (**A**) or SMVET11 (**B**), GC content (black), 6 guinea pig genomes (gray scale), and complete reference genomes from different host and location (colors). The predicted phage regions (black), pSLT (yellow), and IncIγ (gamma) (blue) plasmids are in the outermost ring.

We used PHASTER to examine phage content in all Peruvian *S.* Typhimurium genomes. We found the Gifsy2 (NC_010393) phage present in all Peruvian genomes containing the virulence factor *sodC1*as, expect for *S.* Typhimurium. Whereas the Gifsy-1 (NC_010392) and Sal3 phages were found only in human isolates, we detected five intact (we named these as STGP_Φ1–5) and two questionable (STGP_Φ6 and STGP_Φ7) phage elements in guinea pig isolates (Figure 2A). A STGP_Φ1 phage of ~26 kb was detected in all guinea pig isolates. This phage possesses partial sequence coverage with *Edwardsiella* spp. phage GF-2 (defined by PHASTER), commonly identified in the *S.* Typhimurium DT104 type. Interestingly, pairwise genome alignment using the BRIG tool showed gene content variation in guinea pig genomes driven by the differential presence of prophage sequences (Figure 3). HC50_67462 carried three phages: STGP_Φ3 (~38 kb), STGP_Φ4 (~35 kb), and STGP_Φ6 (~39 kb), absent in other guinea pig genomes. No virulence factor was identified in these phages (Figure 3A); instead, three genomes belonging to the HC50_9757 cluster carried exclusively STGP_Φ5, an intact phage of 42 kb. Finally, one intact (STGP_Φ2) and one partial (STGP_Φ7) phage were present in genomes from the HC50-109967 cluster (Figure 3B). A BLAST search for STGP_Φ7 in the NCBI virus database showed a match with a *Myoviridae* phage of *Escherichia* spp., ESSI2_ev239 (NC_049392.1) with >85% of identity and 76% of coverage. 

## 4. Discussion

In this study, we present a characterization based on the genomic sequences of *Salmonella enterica* subsp. *enterica serovar* Typhimurium isolated from guinea pigs in farms located in Lima-Peru during the period 2015–2016. In Peru, various studies showed the high susceptibility of guinea pigs to infection by *S.* Typhimurium, causing in them a disease characterized by high morbidity and mortality. It is considered the most critical pathogen that affects guinea pig production [16,19,34]. Salmonellosis has been recognized in guinea pigs for several decades, but recently its characteristics have been analyzed at the molecular level [35,36]. A data set of 11 *S.* Typhimurium genomes from guinea pigs were analyzed based on their phylogenetic relationship with other *S.* Typhimurium isolates from humans and various animal species. Additionally, we analyzed the presence of antibiotic resistance genes [ARGs] and mobile genetic elements, like plasmids and prophages.

Our analyzes reveal the presence of two very divergent genetic clusters of *S.* Typhimurium (HC100_9460 and HC100_9757 with ~600 SNPs of difference) circulating in guinea pigs reared in farms located in Lima, Peru. In silico serotype prediction revealed that the HC100_9460 lineage is a monophasic variant of *S.* Typhimurium. Many lineages of monophasic *S.* Typhimurium with different deletion types of the second-phase flagellar genomic region (*fljA, fljB* and *hin* genes) have been reported worldwide [9,37]. The guinea pig HC100_9460 lineage lacks both the *fljA* and *fljB* genes due to the insertion of a phage sequence (STGP_Φ2) but maintains the *hin* gene. In Colombia and Spain, the most prevalent monophasic variants isolated from humans show the full deletion of the *fljAB* operon including the *hin* gene [8,38].

The HC100_9460 and HC100_9757 clusters are found within the phylogroup α, a clade composed of strains from well-characterized epidemics in domestic animals, while clade β harbors lineages that infect wild birds [4,6]. In addition to differential host preferences, clade α isolates contain several lineages with multiple ARGs, while most clade β isolates contain few or no ARGs [6].

Previous studies have used DNA-fingerprinting-based methods to evaluate the genetic variability of *S.* Typhimurium from guinea pigs. The BOX-PCR technique was used in 20 isolates from two farms in Lima in 2015 determining the presence of a single DNA band pattern that suggested a clonal population infecting guinea pigs [35]. In contrast, the ERIC-PCR technique determined at least 7 different patterns in *S.* Typhimurium isolates from guinea pigs of the Peruvian coast collected between 2016 and 2018, showing greater diversity in samples taken in 2018 [39]. Although DNA fingerprinting techniques are difficult to reproduce and show a limited ability to differentiate lineages, our results from genomic data confirm that there are at least two lineages circulating in guinea pig farms in Lima.

On the other hand, the phylogenetic analysis revealed that two (out of five) available genomes from human isolates from Peru were grouped within guinea pig clusters, suggesting the possibility of transmission of *S.* Typhimurium from guinea pigs to humans and vice versa. Although there is no evidence of transmission of *S.* Typhimurium to humans through contact with guinea pigs on farms or at the community level in Peru, a case of salmonellosis was reported in a US family after consumption of guinea pig meat in 2006. It is the first probable association between the consumption of guinea pig meat and human infection for non-typhoidal salmonellosis [18]. In 2017, an outbreak of *Salmonella* Enteritidis was reported in children through exposure to pet guinea pigs in several US states [40]. Although the probability of human salmonellosis infection by contact with pet guinea pigs is rare and unlikely [41], it may be more common in farm environments where a large number of animals and salmonellosis outbreaks are more recurrent, as was described in poultry, pig, and cattle farms [42,43,44]. Although the number of samples in this study limits the ability to predict dominant or more prevalent lineages, the fact that the same lineages have been found in both hosts suggests that these lineages may be epidemiologically important. However, it is necessary to expand the number of guinea pig and human genomes in different geographic regions and over longer periods of time to have a better view of the diversity and evolution of *S.* Typhimurium.

Despite the intensification of guinea pig farming in recent decades, the administration of antibiotics is generally used to treat infections and not as a growth promoter as is commonly used in poultry or pig farms. Therefore, resistance strains are not expected to be found in guinea pigs in the same magnitude as in poultry or pig farms. All *S.* Typhimurium genomes from guinea pigs and humans from the HC100_9757 and HC100_9460 clusters carried the gene for resistance to aminoglycosides *aac(6′)-Iaa*. However, this gene is cryptic, meaning that it does not produce phenotypic resistance [45,46]. In the isolate SMVET 22 from guinea pigs, a resistance genetic cassette was detected containing ARGs associated with resistance to aminoglycoside (*ant(6)-Iaa*), trimethoprim (*dfrA12*), sulfamethoxazole (*sul1*), and quaternary ammonium (*qacE*). 

Aminoglycoside resistance genes are displayed in high frequency in *S.* Typhimurium strains isolated from human feces and animal-based food. Other ARGs frequently described were against tetracyclines, trimethoprim, beta-lactams, fluoroquinolones, and macrolides [47,48]. Resistance to aminoglycosides, trimethoprim, and sulfonamide may be favored by the use of streptomycin, gentamicin, chloramphenicol, nitrofurans, and sulfa-trimethoprim for the treatment of infectious diseases such as salmonellosis in guinea pig colonies in Peru [16,28]. Elevated phenotypic resistance to antimicrobials has been reported in previous studies. Streptomycin [aminoglycoside] resistance was identified in 30% of *S.* Typhimurium isolated from healthy and infected guinea pigs from farms in Lima (Peru) in 2015 [35], whereas sulfamethoxazole resistant strains were found in 68% of isolates from farms of the same city during 2016 and 2018 [39]. Although the use of penicillin is not prescribed in guinea pigs because it is toxic to them [49], strains with a resistant phenotype to ampicillin have been found [35,39]. 

Previous works in *S.* Typhimurium isolates from guinea pigs using PCR detected several ARGs including *qnrB,D* (quinolones), *tetA,B,C* (tetracyclines), and *sul1,2* (sulfamethoxazole), with 23%, 71%, and 57% prevalence, respectively [39], which raises concern about the possible increase in antimicrobial resistance in guinea pig farms. It is interesting that human isolate MOD1-Per91, sampled in 2012, phylogenetically related to the monophasic HC100_9460 lineage from guinea pigs, carried various resistance genes with clinical significance (*floR, fosA3, bla*_CTX-M-65_*, bla*_TEM-1B_, and *mcr*. *1.1* against florfenicol, fosfomycin, extended-spectrum β-lactamases, and colistin). Studies suggest that the transmission of ARGs by horizontal transfer in zoonotic bacteria may be of high risk in the emergence and dissemination of bacteria carrying ARGs between humans and farm animals [50].

Continuous exposure to antibiotics causes stress in bacteria that facilitates the acquisition of ARGs through mobile genetic elements. Plasmids are common mobile elements in *S.* Typhimurium and the tracking is important because they can harbor genes encoding virulence factors, ARGs, and other important genes associated with environment adaptation. In this study, all strains isolated from guinea pigs and humans carried the IncFiB (s) and IncFII (s) plasmids (Figure 2). The FIB (IncFIB) and FII plasmids are commonly found in *S.* Typhimurium (Aljahdali et al., 2020 [51]) and may encode virulence factors and ARGs in *Enterobacteriaceae* species [52,53]. The IncIγ and ColRNAI plasmids were also identified in guinea pigs and human isolates. The IncIγ plasmid is mainly detected in *Escherichia coli* and is associated with horizontal transfer and dissemination of ARGs, such as ESBL (spectrum-extended β-lactamases) and AMPc β-lactamases [54]. ColRNAI is also recognized for harboring ARGs against ampicillin, streptomycin, sulfamethoxazole, and tetracycline, but is present at a low frequency in *S. enterica* [55]. Interestingly, strain SMVET22 carries a plasmid named IncI1 harboring several ARGs (*ant(3′)-laa*, *dfra15*, *qarE* and *sul1*) in a cassette with a transposable element TnAs3. This plasmid has been described strictly in *Enterobacteriaceae*, commonly associated to *Salmonella enterica,* implicated with the spread of ESBL [56], and related to the presence of transposable elements [57]. TnAs3, an insertion element, is abundantly present in plasmids and strongly associated with ARGs and their spread of them in external environments [58].

The identification of phage elements in the genomes of *S.* Typhimurium of Peruvian origin revealed an important diversity of phages. Seven intact [STGP_Φ1–5] and questionable [STGP_Φ6 and STGP_Φ7] phages were identified among the 11 *S.* Typhimurium isolates from guinea pigs, in addition to Gifsy-2, which is found in most isolates of the serovar Typhimurium [6]. The presence of phage sequences in Peruvian isolates shows a lineage-dependent profile suggesting that variation in genetic content is due to dynamic loss/acquisition of phages [59]. In all the guinea pig genomes, the STGP_Φ2 phage was detected, which has a partial match with the *Edwardsiella* spp. bacteriophage GF_2 and is also commonly found in isolates of type DT104 [60], although no virulence genes or ARGs were detected.

The pathogenic ability of *S.* Typhimurium is mediated by genes encoding virulence factors involved in adherence, invasion, intracellular survival, and dissemination. All genomes analyzed in this study were very similar in terms of presence/absence of virulence genes, especially in genomes from clusters HC100_9757 and HC100_9460. The content of genes encoding virulence factors in isolates from humans and guinea pigs suggests that the mechanisms capable of causing infection and disease in both hosts are similar; however, in humans, the infection causes a mild and self-limiting disease [61], unlike the infection in guinea pigs, which causes a disease with high mortality rates and serious injuries to tissues such as the liver. Further studies must be carried out in order to clarify the pathogenesis of salmonellosis in guinea pigs.

In conclusion, the genomic analysis demonstrated the existence of two clades of *S.* Typhimurium circulating among guinea pigs in Lima, including a monophasic *S.* Typhimurium lineage. The close genetic relationship between the guinea pig isolates and some human isolates from Peru found in this study makes it necessary to conduct a more exhaustive investigation to establish possible epidemiological connections between guinea pig and clinical human isolates. The sustained increase in the production of guinea pig meat represents a growing risk to public health due to recurrent outbreaks of salmonellosis in these animals. Future works should consider a greater amount of genomic data from different sources including humans and other animals to determine the impact of *S.* Typhimurium from livestock production systems on human health as well as to monitor the genetic determinants of antimicrobial resistance.

## Figures and Tables

**Table 1:** Genomic statistics of 11 *S.* Typhimurium isolates from guinea pigs in Lima, Peru.

Isolate	Biosample ID	Average Coverage	Number of Contigs	Number of Bases	Number of CDS	%GC	MLST	Completeness *	Contamination *	HierCC HC100
SMVET11	SAMN28944802	61.012	44	4,851,410	4514	52.23	19	100	0.39	9460
SMVET12	SAMN28944803	76.632	45	4,902,895	4571	52.14	19	100	0.08	9757
SMVET19	SAMN28944804	93.268	61	5,088,465	4780	52.08	19	100	0.39	9757
SMVET20	SAMN28944805	106.307	39	4,849,526	4514	52.23	19	100	0.39	9460
SMVET21	SAMN28944807	83.118	44	5,020,376	4699	52.10	19	100	0.08	9757
SMVET22	SAMN28944808	115.677	54	5,095,938	4803	52.07	19	100	0.08	9757
STc10	SAMN28944806	30.112	175	5,046,798	4725	52.22	19	100	0.08	9757
STc12	SAMN28944811	36.224	65	5,017,046	4694	52.13	19	100	0.08	9757
STc8	SAMN28944810	31.676	80	4,915,140	4563	52.09	19	99.69	0.15	9757
STc9	SAMN28944809	61.838	39	4,891,950	4548	52.15	19	100	0.08	9757
VET1	SAMN28944812	316.099	86	4,910,420	4557	52.12	19	100	0.9	9757

* Values calculated by CheckM software.

## Data Availability

The genome data presented in this study are publicly available in NCBI with the accession numbers SAMN28944802, SAMN28944803, SAMN28944804, SAMN28944805, SAMN28944806, SAMN28944807, SAMN28944808, SAMN28944809, SAMN28944810, SAMN28944811, SAMN28944812.

## Supplementary Materials

The following supporting information can be downloaded at: https://www.mdpi.com/article/10.3390/microorganisms10091726/s1, Table S1: List of genomes sequenced and information about farms, locations, and year of isolation; Table S2: List of genomic sequences downloaded from Genbank used as context for the phylogenomic analysis; Table S3: Incompatibility plasmid, ARGs, and prophages profiles found in S. Typhimurium isolates from guinea pigs and humans; Figure S1: SNP distance matrix of *S*. Typhimurium genomes from guinea pigs and the LT2 reference strain. Color gradients indicate the number of SNPs between isolates from lowest to highest, as shown in the legend, while the number within each square represents the number of SNPs between two genomes. Dendrograms were constructed using hierarchical clustering in the *pheatmap* package; Figure S2: Scheme that represents gene deletion patterns in the *fljAB* operon. From above, the intact structure of the *fljAB* operon of the LT2 strain, followed by the intact structure of the *fjlAB* operon of the SMVET21 strain belonging to the H100_9757 cluster. Below, the *fljAB* operon detected by the STGP_Φ2 phage of the monophasic strain SMVET11 belonging to the H100_9460 cluster; Figure S3: SNP distance matrix of 37 *S*. Typhimurium genomes used in this work. Color gradients indicate the number of SNPs between isolates from lowest to highest, as shown in the legend, while the number within each square represents the number of SNPs between two genomes; Figure S4: Repertoire of virulence genes in *S.* typhimurium strains. The green square indicates the presence of the gene while the white indicates absence.
