# Supplementary figures and images for: Genomic Characterization of Salmonella Typhimurium Isolated from Guinea Pigs with Salmonellosis in Lima, Peru
Source: Microorganisms. 2022 Aug 27;10(9):1726. doi: 10.3390/microorganisms10091726 (PMC9503038; doi:10.3390/microorganisms10091726)

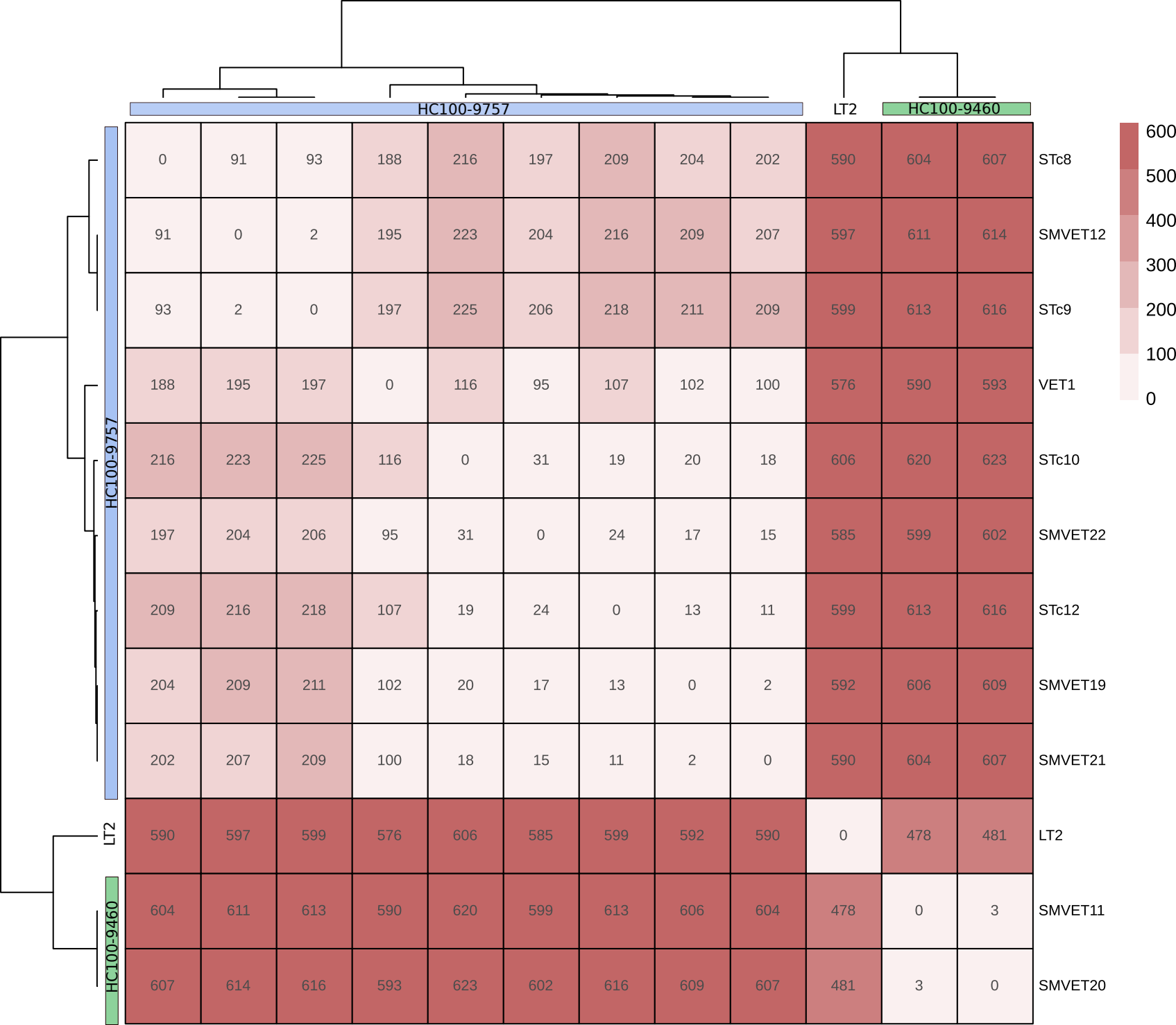

Supplement: Supplementary file 1 [file microorganisms-10-01726-s001.zip › Figure S1.pdf]

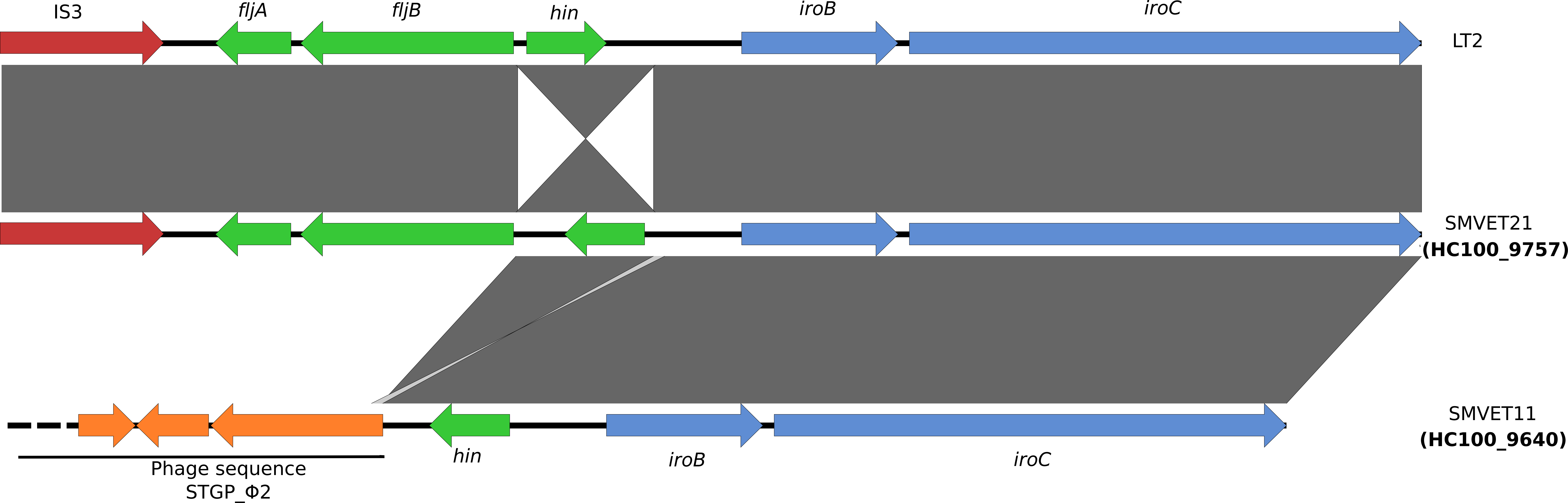

Supplement: Supplementary file 1 [file microorganisms-10-01726-s001.zip › Figure S2.pdf]

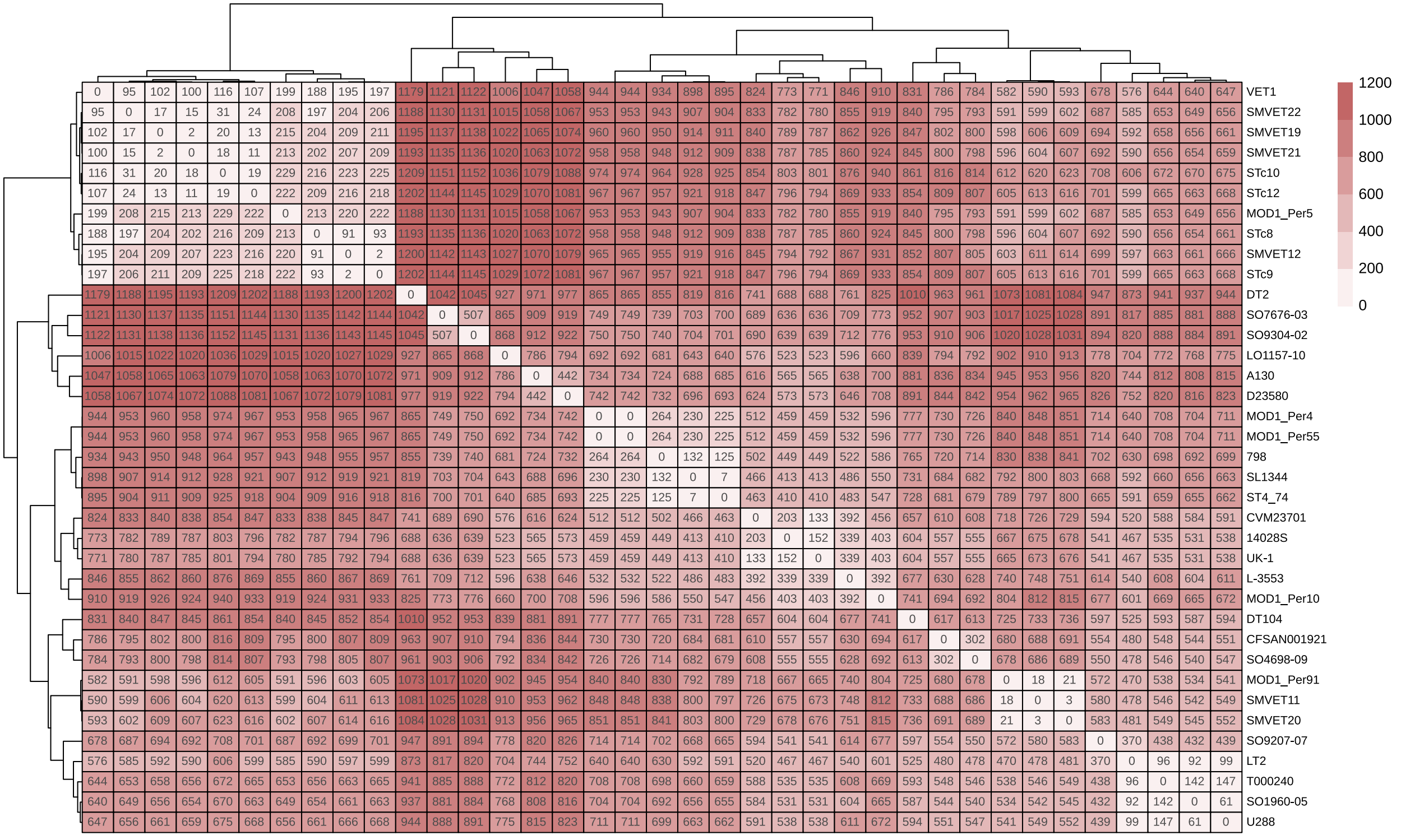

Supplement: Supplementary file 1 [file microorganisms-10-01726-s001.zip › Figure S3.pdf]

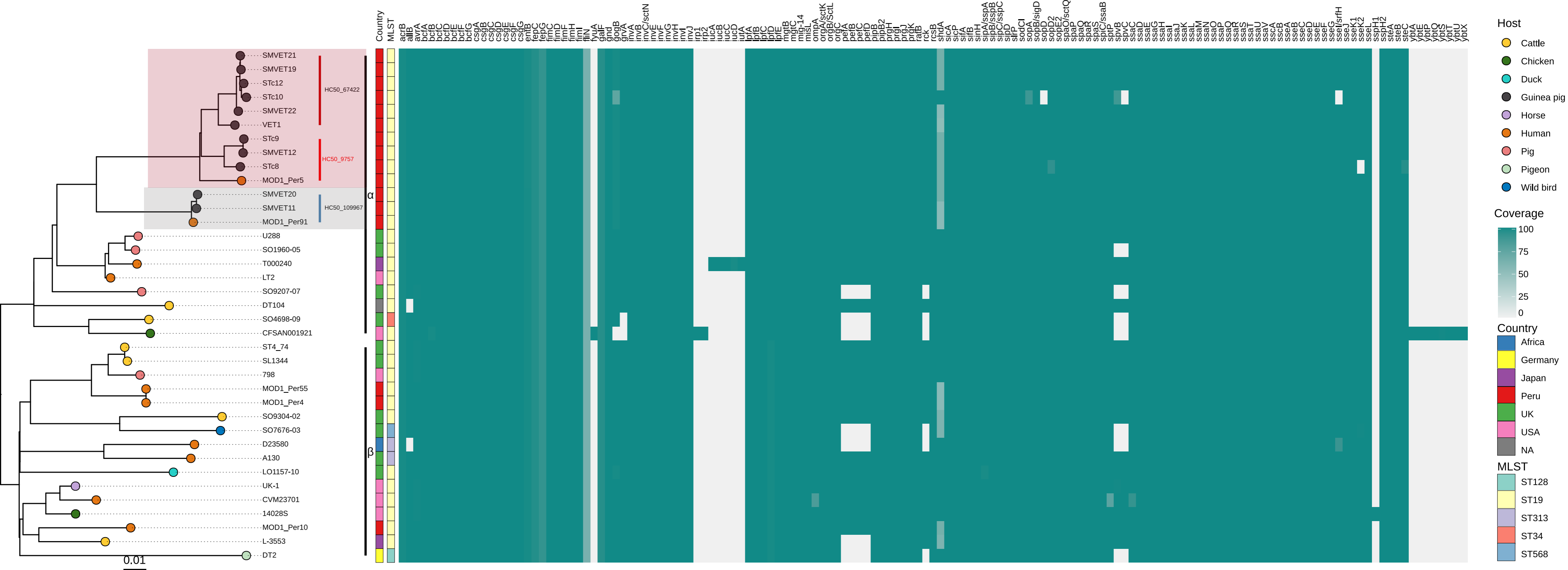

Supplement: Supplementary file 1 [file microorganisms-10-01726-s001.zip › Figure S4.pdf]
